# Native electrospray mass spectrometry approaches to probe the interaction between zinc and an anti-angiogenic peptide from histidine-rich glycoprotein

**DOI:** 10.1038/s41598-018-26924-1

**Published:** 2018-06-05

**Authors:** Esther M. Martin, Frances D. L. Kondrat, Alan J. Stewart, James H. Scrivens, Peter J. Sadler, Claudia A. Blindauer

**Affiliations:** 1 Department of Chemistry, University of Warwick, Coventry, UK; 2 Medimmune, Cambridge, UK; 3 School of Life Sciences, University of Warwick, Coventry, UK; 4 Immunocore Ltd, Abingdon, UK; 5 School of Medicine, University of St Andrews, St Andrews, UK; 6 School of Science, Engineering and Design, Teeside University, Middlesbrough, UK

## Abstract

Zinc modulates the biological function of histidine-rich glycoprotein (HRG) through binding to its His-rich region (HRR). The Zn^2+^-binding properties of a 35 amino-acid biologically-active peptide mimic of the HRR, HRGP330, were investigated using dissociative mass spectrometry approaches in addition to travelling-wave ion mobility mass spectrometry (TWIM-MS). Native mass spectrometry confirmed zinc binding to HRGP330; however, broadening of the ^1^H NMR resonances upon addition of Zn^2+^ ions precluded the attainment of structural information. A complementary approach employing TWIM-MS indicated that HRGP330 has a more compact structure in the presence of Zn^2+^ ions. Top-down MS/MS data supported a metal-binding-induced conformational change, as fewer fragments were observed for Zn^2+^-bound HRGP330. Zn^2+^-bound fragments of both N-terminal and C-terminal ends of the peptide were identified from collision-induced dissociation (CID) and electron transfer dissociation/proton transfer reaction (ETD/PTR) experiments, suggesting that multiple binding sites exist within this region of HRG. The combination of mass spectrometry and NMR approaches provides new insight into the highly dynamic interaction between zinc and this His-rich peptide.

## Introduction

Histidine-rich peptides, loops and regions are ubiquitous in the proteomes of all organisms^1,2^. They are found in antimicrobial histatins^3^, snake venoms^4^, proteins involved in metal ion homeostasis^5^, and proteins mediating interactions with anionic molecules or surfaces^6^. There are no resolved 3D structures available for any His-rich region; invariably such regions have proven to be refractory to classical structural analysis, most likely due to structural disorder^7^. Indeed, His-rich proteins and peptides are one prominent example of intrinsically disordered proteins^6^.

**Figure 1:**
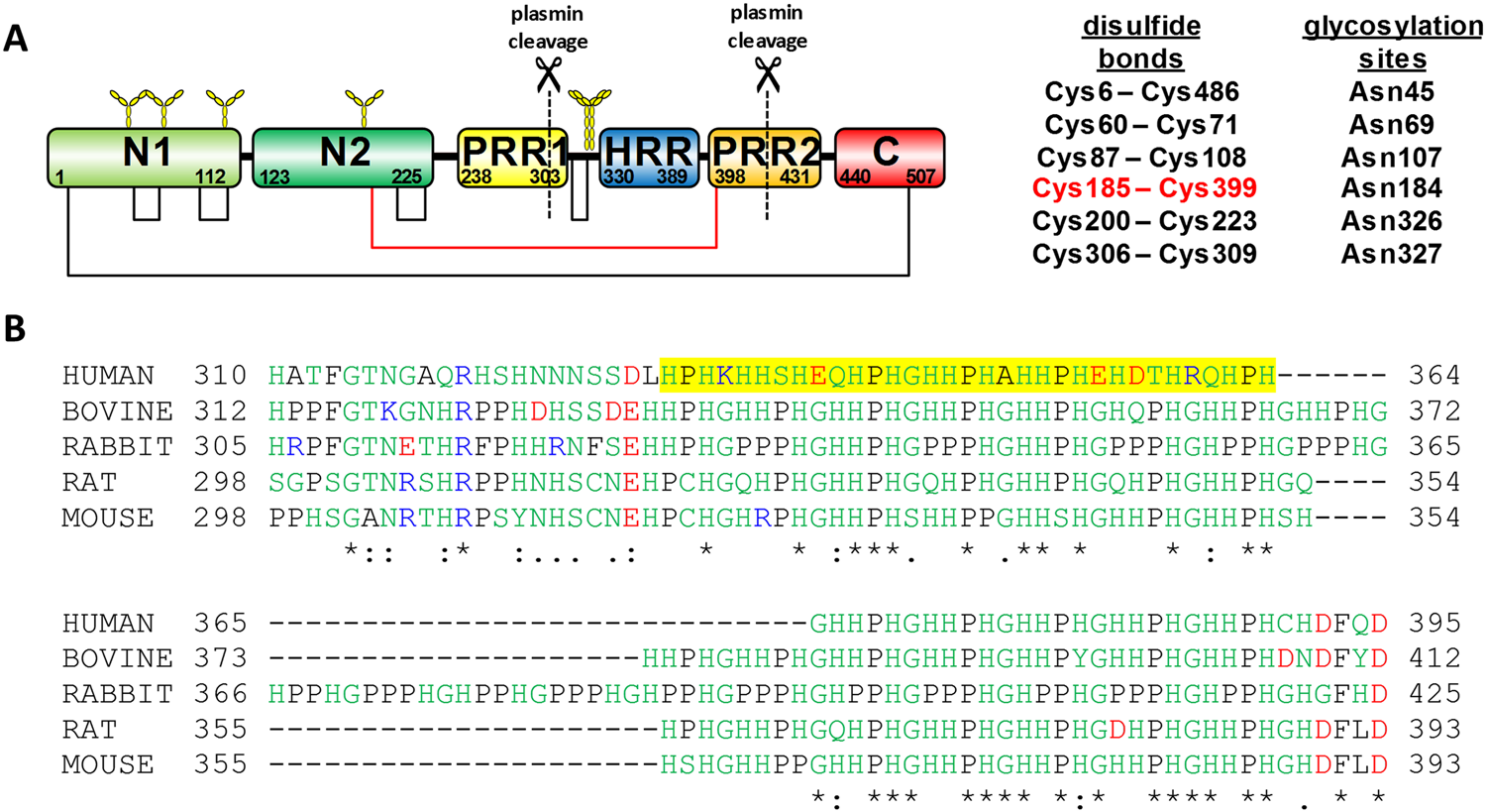
Domain structure and HRR sequences of HRG. (**A**) Domain structure of human HRG showing the disulfide bridging arrangement and six putative glycosylation sites. The disulfide bridge between the N2 domain and the fragment which can be released by plasmin-mediated cleavage is highlighted in red. Notably, this fragment contains the entire HRR. (**B**) Sequence alignment of the HRR of HRG from various mammalian species. The sequence of the 35-residue HRGP330 peptide is highlighted in yellow. Amino acids are coloured according to their chemical properties: hydrophobic (black), acidic (red), basic (blue), hydrophilic (green). Symbols represent conservation of amino acids: fully conserved (*), conservation between groups with strongly similar properties (:) and conservation between groups of less similar properties (.). The sequence for rabbit HRG, which has been recently amended11, corresponds to NCBI reference sequence XP_008264798.1.

An important property of His-rich proteins and peptides is their ability to bind metal ions, and in many cases, this ability relates to their biological function. In organisms, they are predominantly associated with Ni^2+^, Cu^2+^, and Zn^2+^ ^1^. One particularly intriguing example is the His-rich region (HRR) from mammalian histidine-rich glycoproteins (HRGs). Human HRG is a 75 kDa plasma protein^8^ that was first isolated from human serum in 1972^9^. It is synthesised in the liver and is present in plasma at a relatively high concentration (*ca*. 1.5–2 µM). HRG possesses a multi-domain structure, consisting of six functional domains (Fig. 1A)^10,11^. These include two N-terminal regions, N1 and N2, which display homology to cystatins. The X-ray crystal structure of the N2 domain was recently reported, confirming that it exhibits a cystatin-like fold^12^. The HRR (Fig. 1B) contains repeating units of GHHPH pentapeptides, and is flanked at either side by Pro-rich regions (PRR). Finally, there is a C-terminal domain. Four intradomain and two interdomain disulfide bonds as well as six predicted N-glycosylation sites at various Asn residues are distributed across the protein^1,13^.

HRG is able to interact with many ligands^14,15^ including proteins such as plasminogen^16^, fibrinogen^17^, thrombospondin-1 (TSP-1)^18^, vasculostatin^19^, and immunoglobulins^20^. Other binding partners include heparins and heparan sulfate^21^, heme^22,23^, and metal cations such as Cu^2+^ and Zn^2+^ ^24^. In this context, the HRR is a particularly interesting part of the protein, as not only are the imidazole nitrogens of this region thought to play a major role in Zn^2+^ binding, but the latter has also been shown to enhance many of the reported HRG-ligand interactions, at physiologically relevant concentrations^25–30^.

HRG is involved in the regulation of numerous biological processes including coagulation, immune complex clearance and angiogenesis, processes which have to be tightly controlled. Unregulated angiogenesis, the process by which new blood vessels are formed after injury, is associated with pathological conditions including ischemia, rheumatoid arthritis and cancer^31^. Indeed, the design and use of molecules that inhibit angiogenesis constitutes an important therapeutic strategy in cancer treatment. It has been proposed that the HRR region of HRG may be proteolytically released from HRG by plasmin (as shown in Fig. 1A) to give rise to a fragment with anti-angiogenic properties^32^. Our recent identification of an *in vivo S*-glutathionyl adduct at Cys185 of the N2 domain of rabbit HRG supports this hypothesis^12^. Cys185 usually forms a bridged disulfide bond with a cysteine residue in this region (Cys407 in rabbit HRG) that must be cleaved to allow plasmin-mediated release of the HRR-containing fragment from the intact protein.

Significantly, even shorter segments of the HRR have shown anti-angiogenic activity^33^. HRGP330 is a 35 amino acid peptide derived from the HRR of human HRG (Fig. 1B). Its primary sequence consists of amino acids 330–364. HRGP330 at a concentration of 100 ng/mL was able to inhibit chemotaxis of primary endothelial cells following stimulation by vascular endothelial growth factor. It is thought that the interaction of this and other HRG-derived peptides is mediated by binding to cell surface heparan sulfate^34^. Importantly, these interactions are enhanced by the presence of Zn^2+^. Likewise, the broad-spectrum antimicrobial activities of HRG and HRG-derived His-rich peptides are also Zn^2+^ dependent^13^. These findings suggest that peptides which correspond to particular sections of the HRR mimic some of the molecular properties associated with HRG or its large proteolytic fragment. Moreover, given that the peptides themselves exhibit Zn^2+^-dependent anti-angiogenic activity and have potential therapeutic value, their interaction with Zn^2+^ ions is of immediate interest. We therefore initiated studies on Zn^2+^ complexes of HRGP330.

Questions that we sought to address include (i) how many zinc ions can be bound by the peptide? (ii) does zinc binding have an impact on structure? and (iii) is it possible to identify individual zinc binding sites? In the present study, we will show how native electrospray mass spectrometry (nESI-MS), travelling wave ion mobility mass spectrometry (TWIM-MS), and top-down mass spectrometry, in combination with ^1^H NMR spectroscopy, substantially contribute towards answering all three questions.

## Results

### Native mass spectrometry identifies multiple Zn-bound species

nESI-MS allows study of intact biomolecules in states that are typically close to the native fold^35–37^. In contrast to standard mass spectrometry conditions, this involves working with aqueous solutions at around neutral pH, with little or no organic solvent added. Crucially, this allows preservation of the protein tertiary structure as well as non-covalent interactions, and thus protein-ligand complexes can be investigated^38^. This includes interactions with essential metal ions such as Zn^2+^ ^39–44^. nESI-MS is the only method providing direct and simultaneous information on stoichiometry and speciation of metal-protein complexes present in a mixture, having the unique ability to report on each individual species present^45–47^.

**Figure 2:**
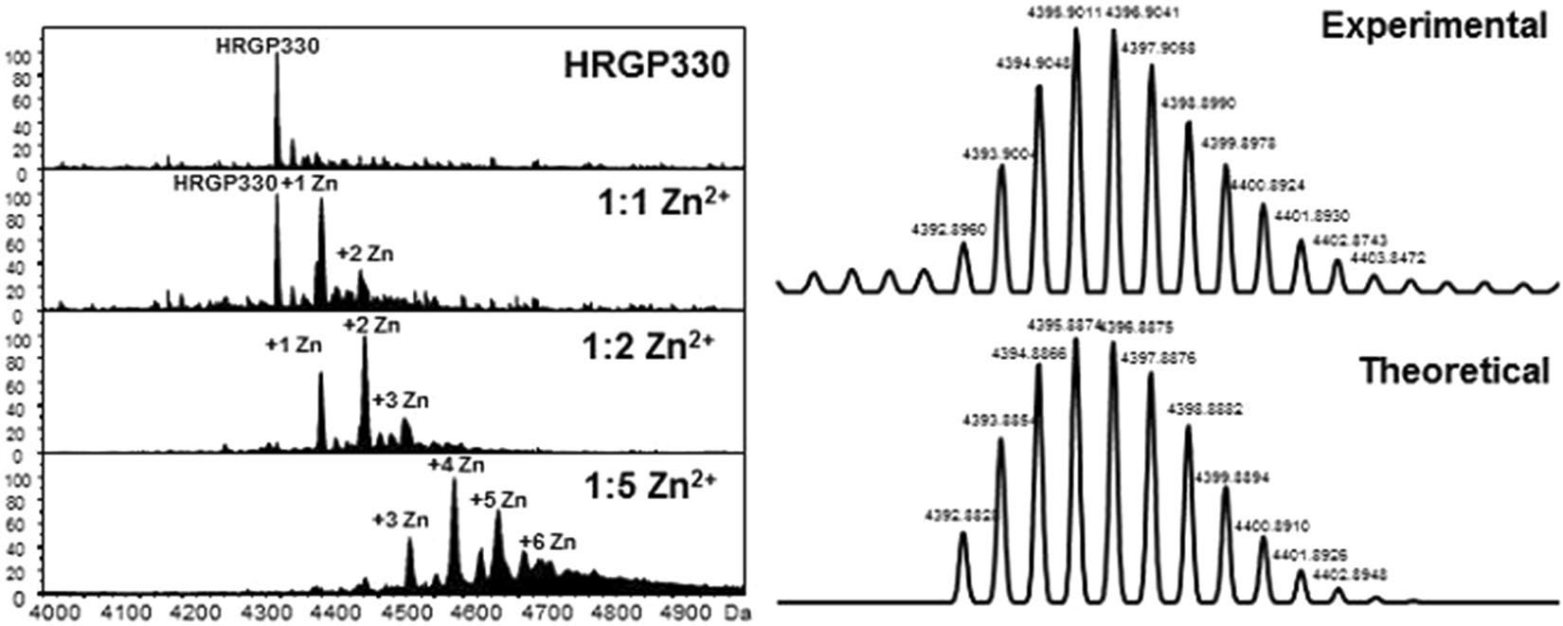
Deconvoluted ESI-MS spectra of HRGP330 incubated at varying Zn^2+^:peptide ratios. Peptide samples (15 μM) in 10 mM ammonium acetate (pH 7.40) were titrated with zinc acetate and were analysed on a maXis QToF mass spectrometer (Bruker Daltonics). The isotopic distribution for the 1:1 Zn-HRGP330 complex is shown on the right hand side together with a comparison to the theoretical model.

Crude synthetic HRGP330 was purified by Reverse-Phase-HPLC (Supplementary Fig. S1) and its identity was confirmed by ESI-MS. An experimental average neutral mass of 4333.98 Da was determined, within 1 Da of the theoretical average neutral mass of 4334.58 Da (Supplementary Fig. S2). HRGP330 was incubated at varying Zn/peptide molar ratios and then analysed by nESI-MS. Figure 2 shows the resulting deconvoluted mass spectra. These indicate that several different HRGP330-metal species are formed. At a HRGP330:Zn^2+^ ratio of 1:1, Zn_1_-HRGP330 was observed at the same intensity as the peak for apo-HRGP330. A small amount of Zn_2_-HRGP330 was also present at this stage. The latter form was the most abundant at 2 molar equivalents (mol. eqs.) of Zn^2+^, and the apo-form had almost completely disappeared. When 5 mol. eqs. of Zn^2+^ were present in the reaction mixture, a range of HRGP330-Zn species were observed. The Zn_4_ form was the most abundant, followed by Zn_5_ and Zn_3_, whilst the Zn_6_ and Zn_2_ forms were also identifiable in very low amounts. In its 35-aa sequence, HRGP330 contains 17 His and 4 Glu or Asp residues, i.e. up to 21 residues with appreciable potential for metal binding. Assuming a requirement for at least four ligands per Zn^2+^ and only mono-dentate binding would thus allow for up to 5 Zn^2+^ ions to be bound. The occurrence of the Zn_6_ form at the 5:1 ratio suggests that the maximal binding capacity of the peptide may involve either further residues (e.g. glutamines), the possibility of aspartates and glutamates acting as bridging ligands, or the population of binding sites with less than four peptide-derived ligands.

### Zinc binding severely broadens ^1^H NMR signals of HRGP330

**Figure 3:**
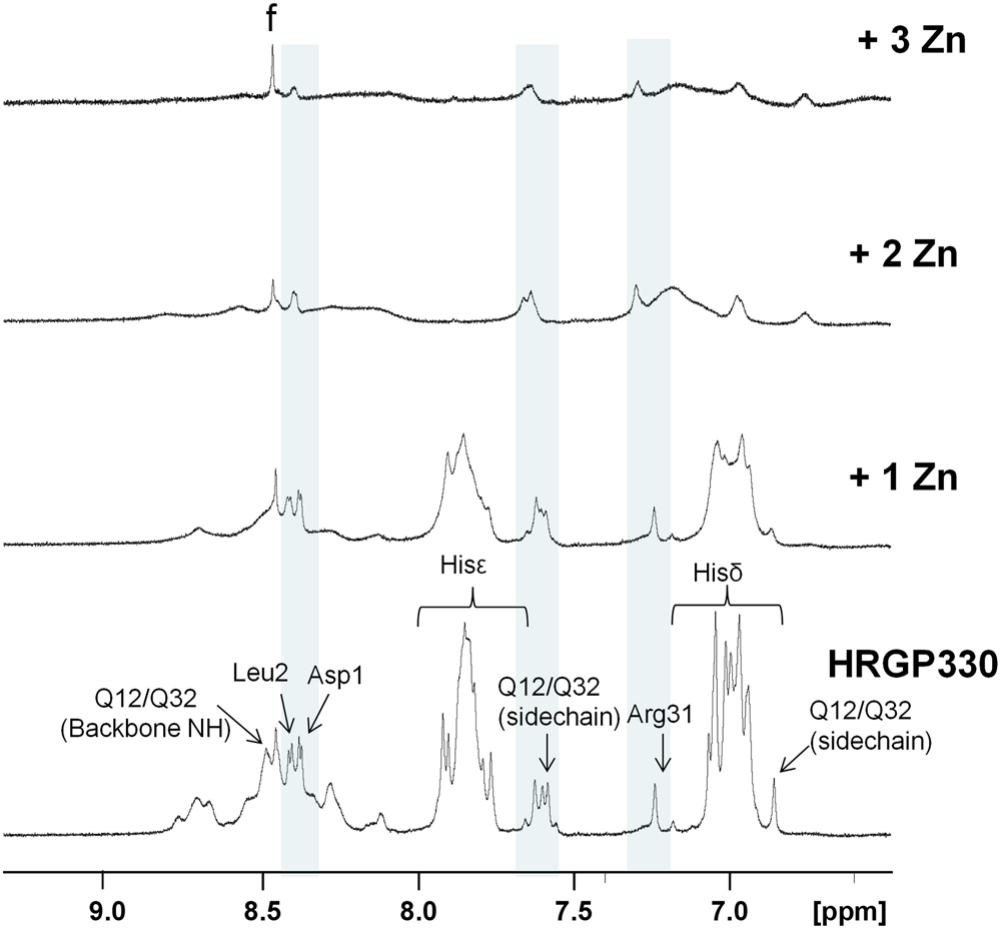
Stacked plot of 700 MHz 1D ^1^H-NMR spectra showing the fingerprint region of HRGP330 in the presence of varying amounts of ZnCl_2_. 0.5 mM samples were prepared in 50 mM Tris[D_11_], 50 mM NaCl with 10% D_2_O at pH* 6.2 and 278 K. Aromatic protons from His residues are annotated, but significant overlapping of peaks meant they could not be assigned unambiguously in 2D spectra (Figs S3 and S5). A peak corresponding to formate (chemical shift standard) is denoted by “f”.

^1^H NMR spectroscopy was used to study qualitatively the effects of zinc binding on structure and dynamics of HRGP330. The 1D and 2D ^1^H NMR spectra of apo-HRGP330 (Figs 3 and S3) were relatively well-resolved, but showed limited dispersion of resonances. The closeness of observed ^1^H chemical shifts of backbone protons to so-called random-coil shifts indicates that no regular secondary or tertiary structure is adopted. This is consistent with the idea that HRGP330 is an example of an intrinsically disordered peptide, which is also supported by the appearance of its far-UV CD spectrum (Supplementary Fig. S4). From their aromatic cross-peaks, at least 11 His residues were distinguishable, but only very limited sequential assignment was possible due to the lack of dispersion.

Upon addition of 1 mol. eq. of ZnCl_2_ both aromatic side-chain (from His) and backbone NH resonances showed considerable line broadening (Fig. 3). No new peaks were observed across the spectrum upon addition of Zn^2+^. Inspection of 2D TOCSY NMR data for this sample (Supplementary Fig. S5) revealed that all imidazole cross-peaks were already broadened beyond detection at this molar ratio, whilst the side-chain amide proton signals of Gln residues, as well as the proton signals of most aliphatic side-chains, were still readily detectable. The fact that all His side-chains were equally affected may suggest that there is no clearly defined preferential binding site. In addition, all previously observed backbone amide resonances, with the exception of Asp1 and Leu2, had broadened beyond detection, a typical sign of configurational and/or conformational fluctuations on an intermediate timescale. After addition of 2 mol. eq. ZnCl_2_, even more severe line-broadening was observed, and when higher metal:peptide ratios were used, the overall signal-to-noise ratio decreased markedly, indicative of a loss of peptide in solution. Indeed, at these higher concentrations of Zn^2+^, extensive aggregation of the peptide was visible as a precipitate in the NMR tube. This is likely a consequence of the relatively high concentrations used in the NMR experiment, as only a small amount of dimer (~1.5 % relative intensity, not shown) and no higher oligomers were observed in samples analysed by ESI-MS. It may be assumed that the oligomers and aggregates involve inter-peptide Zn bridges.

Therefore, due to generally low spectral dispersion, coupled with significant line broadening even at the lowest Zn^2+^ ratio, and oligomerisation and precipitation at higher Zn^2+^ concentrations, no further structural information on the HRGP330-Zn complexes could be gleaned from the NMR experiments. The behaviour in NMR experiments exhibited by HRGP330 upon zinc addition is quite typical for metal-binding peptides interacting with Zn^2+^ ^48^. The disappearance of backbone NH resonances from a relatively small peptide can be caused by several processes, including oligomerisation and intermediate exchange between multiple species. Based on the loss of signals at higher Zn^2+^ ratios as well as on the ESI-MS data shown in Fig. 2, it can be suggested that in the present case, both processes may be involved in causing this observation. We therefore employed TWIM-MS to obtain global structural information on several individual zinc-bound HRGP330 species, under conditions disfavouring oligomerisation and aggregation.

### Conformational diversity of HRGP330 increases upon zinc binding

nESI-MS becomes an even more powerful technique when combined with Ion-Mobility-MS (IM-MS)^35^. Here, the addition of a gas-filled mobility cell allows separation of ions by their size, as more compact ions travel through the gas at higher velocity^49^. IM-MS can provide insight into the overall shape of the molecules studied, with more compact conformers interacting less with the mobility gas than more extended/unfolded conformers. In the case of proteins and peptides, this relates to folding, with more compact conformers travelling faster than more extended/unfolded conformers. IM-MS has been applied successfully to metalloproteins^43,44,50–57^, and has been shown to give information on protein conformation that is complementary to other more traditional techniques. IM-MS is particularly useful in cases where the molecules under study are not amenable to classical structural analysis^58,59^, such as encountered in the present case.

**Figure 4:**
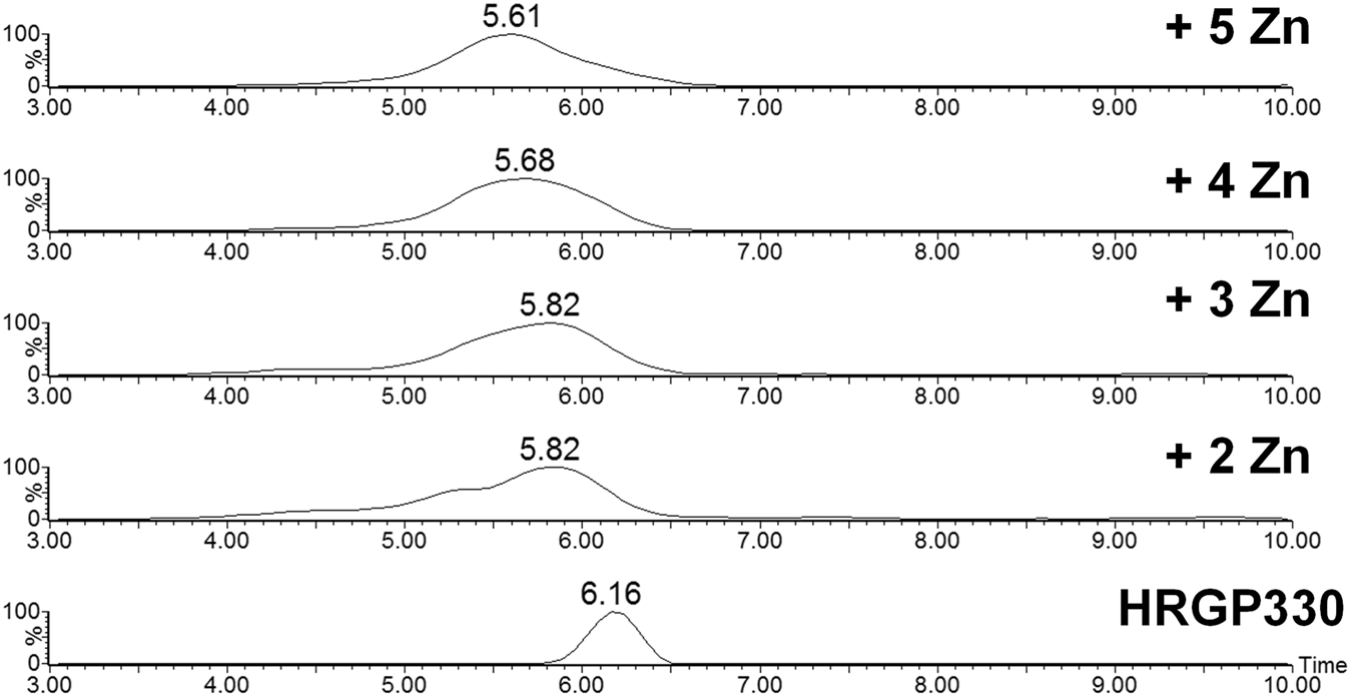
Effect of Zn^2+^ binding on the ATD of the 5+ charge state of HRGP330. ATD peaks of Zn^2+^ bound species, given in ms. The sample consisted of 10 µM HRGP330 in 10 mM ammonium acetate (pH 7.4) and 50 μM Zn. The Zn_1_-HRGP330 peak identified in the sample was too low in intensity to give reliable data. The trace for the apo-peptide was acquired on a zinc-free sample. ATD data for all charge states for apo-HRGP330 are shown in Fig. S6.

Therefore, TWIM-MS (Travelling-wave ion mobility mass spectrometry) was used to examine how Zn^2+^ affects the conformation of the HRGP330 peptide. A comparison of the arrival-time distributions (ATD) for the 5+ charge state of apo-HRGP330 and the various Zn^2+^-peptide complexes is shown in Fig. 4.

Apo-HRGP330 produced a narrow peak distribution, whilst all four zinc-bound species (detected in a single sample) showed much broader ATDs. Since ATDs are correlated to collisional cross-sections, a broader ATD is usually interpreted as an indication for the presence of a wider range of conformers; in the present case, it is likely that configurational isomers, with Zn^2+^ ions bound in different locations, also contribute to this observation. It is also significant that the average arrival time decreased upon zinc binding, indicating that the zinc-bound conformers were on average more compact than the apo-form. This is a commonly observed behaviour, even for non-specific complexes of metal ions with small peptides^60^. Thus, although the observed compaction in the gas phase is consistent with the idea that zinc binding restricts the flexibility of the peptide, it is insufficient evidence to postulate a specific zinc-induced conformational change.

### Zinc binds to multiple sites in Zn_1_-HRGP330

Considering that under basal physiological conditions, HRG in blood plasma, and by inference HRGP330, is expected to have not more than one molar equivalent of Zn^2+^ bound^30^, it was of interest to investigate further how HRGP330 interacted with an equimolar amount of Zn^2+^. Since ^1^H NMR spectroscopy was not able to give detailed structural insights, we explored the utility of tandem MS methods to locate Zn^2+^ ions on HRGP330.

Tandem MS approaches have previously been used to pinpoint preferred interaction sites of metal ions or their complexes on proteins. The majority of such studies concern protein modifications with 2^nd^ or 3^rd^ row transition metal complexes, especially in the context of anti-cancer metallodrugs based on ruthenium or platinum^61–67^. Typically, the bonds that such metal ions form with protein side-chains are strong, have significant covalent character, and often are kinetically quite inert – hence, the experimental conditions that can be employed in a respective MS experiment can be comparatively harsh, both in terms of proton concentration (availability of H^+^ enhances ionisation efficiency in positive ESI-MS) and fragmentation technique. This is in contrast to a metal ion such as the 1^st^ row d-block Zn^2+^, which binds to protein side-chains not only less strongly, but also in a much more kinetically-labile fashion, and with bonds usually being very susceptible to proton-induced dissociation. This is the case for HRGP330: Below pH 5, very little zinc is bound to the peptide, even when present at a 5-fold excess (Supplementary Fig. S7). Therefore, it is not trivial to maintain zinc binding during ionisation and fragmentation in the mass spectrometer. Top-down MS under native conditions, especially in conjunction with alternative fragmentation techniques, may offer opportunities to tackle these challenges. Non-covalent protein assemblies have previously been analysed by MS/MS experiments under (near-)native conditions^68–70^, and examples involving interactions between Zn^2+^ and proteins/peptides also exist^40,71–74^. In addition, MS/MS studies on the interactions between peptides such as angiotensins and M^2+^ ions are also available under non-native and excess metal conditions^39,75,76^.

Native mass spectra of apo-HRGP330 and a 1:1 zinc complex of HRGP330, prior to isolation and fragmentation, are shown in Supplementary Fig. S8. Both species were subjected separately to collision-induced dissociation (CID) and electron-transfer dissociation/proton transfer reaction (ETD/PTR) fragmentation. CID typically cleaves the peptide bonds, and produces predominantly *b* and *y* ions (Biemann notation) whereas ETD is a radical-driven fragmentation technique which generates a series of *c* and *z* ions following cleavage of N-Cα bonds. ETD can be implemented on a range of MS instruments and is, like the FT-ICR-MS (Fourier-Transform Ion Cyclotron Resonance Mass Spectrometry) based electron capture dissociation (ECD^77^, thought to be a non-ergodic process^78^. As a consequence, weak interactions may be preserved during fragmentation. For this reason, ECD and ETD are particularly valuable for the identification and localisation of post-translational modifications^79^. ETD can be followed by proton transfer reaction (PTR) which decreases the charge states of the ETD-fragments produced, thus simplifying the resulting spectra and allowing easier peak assignment.

**Figure 5:**
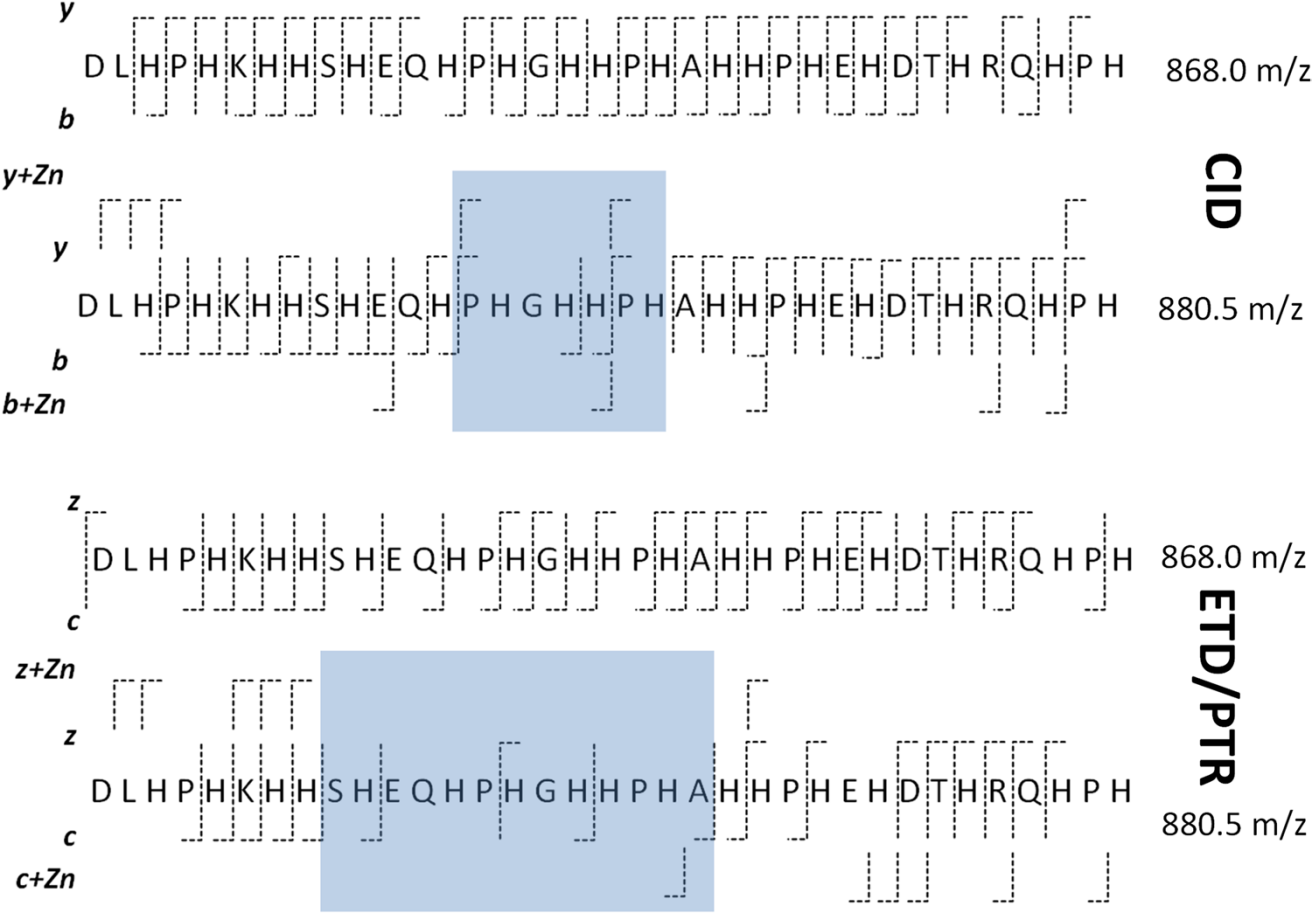
Fragmentation of the +5 charge states of apo (868.0 m/z) and Zn_1_-HRGP330 (880.5 m/z) by CID and ETD/PTR. Fragmentation schemes are derived from analysis of spectra shown in Supplementary Figs S9–S11 and S13, using Biotools v3.2 and Sequence Editor v3.2, or manual peak assignments in the case of ETD/PTR for 880.5 m/z, where only weak fragment peaks were obtained. The boxes shaded in grey indicate areas where cleavage efficiency decreased dramatically upon zinc binding.

In both fragmentation approaches and for both apo-HRGP330 and the Zn_1_-HRGP330 complex, the 5+ charge state was selected for fragmentation as this showed the greatest intensity (Supplementary Fig. S8), and also because higher charge states are more favourable for fragmentation by ETD. For the metal-free peptide, the 868.0 m/z ion ([M+5H]^5+^) was isolated and fragmented using CID and ETD. The annotated product spectra are shown in Figs S9 and S10. The Biemann nomenclature was used to annotate the fragments, and fragmentation schemes in Fig. 5 summarise the cleavages that occurred in each experiment.

Good sequence coverage was achieved for apo-HRGP330 in both experiments: 82.9% in the ETD fragmentation compared to a slightly lower value of 74.6% for CID. This is a common observation; CID often gives rise to lower sequence coverage than ETD because ETD typically produces a well-defined ion series, whilst CID cleavage efficiency is more sequence-dependent^79,80^. Yet, full sequence coverage cannot be expected for ETD because HRGP330 contains 5 Pro residues, and due to the cyclic structure of prolines, cleavage of their N-Cα bond does not lead to dissociation of the peptide.

In the case of zinc-bound HRGP330, the 880.5 m/z peak ([HRGP330+Zn+3H]^5+^) was selected for fragmentation. CID of Zn^2+^-bound HRGP330 produced a larger number of fragment ions than CID of apo-HRGP330, suggesting that additional fragments with Zn^2+^ ions bound were potentially formed (Supplementary Fig. S11). Indeed, a number of Zn^2+^-bound fragments were identified from their characteristic isotopic distributions (Fig. S12). Up to approximately 700 m/z, the CID spectra of 868.0 m/z (apo) and 880.5 m/z (Zn_1_) did not differ significantly, indicating that no Zn^2+^ remained bound to the respective smaller (<b_6_) or more highly charged and smaller (<y_11_^2+^ or b_11_^2+^) fragments. Table 1 summarises the Zn^2+^-bound fragments that were identified and their corresponding Zn^2+^-free fragments (as observed for the apo form).

**Table 1:** Summary of Zn^2+^-bound peptides identified from CID, together with experimental and theoretical masses for the respective parent fragment ions.

Assignment of fragment	Experimental [M+H]^1+^	Theoretical [M+H]^1+^	∆mass (Da)	Experimental [M-H+Zn]^1+^	Theoretical [M-H+Zn]^1+^	∆mass (Da)
y_8_	1026.68	1026.49	+0.19	1087.54	1087.40	+0.14
y_10_-NH_3_	1275.70	1275.57	+0.13	1337.74	1337.50	+0.24
b_11_	1397.70	1397.64	+0.06	1459.65	1459.55	+0.10
y_12_	1526.96	1526.71	+0.25	1588.86	1588.62	+0.24
y_17_	2106.34	2105.97	−0.37	2168.20	2167.89	−0.31
b_18_	2290.08	2289.93	+0.15	2290.08	2289.93	+0.15
y_22_	2671.30	2671.22	+0.08	2733.24	2733.14	+0.10
b_23_	2807.32	2807.28	+0.04	2869.24	2869.19	+0.05
b_28_	3422.68	3422.52	+0.16	3484.54	3484.43	+0.11
y_29_	3563.74	3563.60	+0.14	3625.63	3625.51	+0.12
y_32_	3926.15	3925.80	+0.35	3988.15	3987.71	+0.44
y_32_-H_2_O	3908.12	3907.79	+0.33	3970.00	3969.71	+0.29
y_33_	4125.12	4124.77	+0.35	4125.12	4124.77	+0.35
b_33_	4082.02	4081.84	+0.18	4144.04	4143.76	+0.28
y_34_	4176.65	4175.94	+0.71	4238.30	4237.86	+0.44
y_34_-NH_3_	4158.60	4158.92	−0.32	4220.65	4220.85	−0.20

Many of these are relatively large fragments and do not provide specific information on the location of any preferred binding site(s). The smallest fragment identified to bind Zn^2+^ was y_8_, which suggests that in this fragment, the metal ion is interacting with His35, His33, and/or His27. This may indicate that at least three His residues are required for sufficiently stable Zn^2+^ coordination. In addition, fragments from the N-terminal end of the molecule were also observed as Zn^2+^ complexes. Here, the smallest fragment was b_11_ which indicates that any of the five His residues (plus Asp1 and Glu11) in the sequence D^1^LHPHKHHSHE^11^ could be coordinating to a Zn^2+^ ion.

Whilst the CID spectrum of the zinc-bound species (m/z = 880.5) showed a significant amount of fragmentation which overall was similar to that observed for the apo species, the ETD spectrum (Figs 5 and S13) was dominated by unfragmented Zn-HRGP330 in its +2 and +3 charge states. Fragment peaks were much less intense, not well resolved, and also less numerous. This indicates that in the case of this His-rich peptide, complexation with Zn^2+^ had a considerable negative impact on overall fragmentation efficiency. In the light of literature findings showing that positively charged, non-redox-active ions support or enhance fragmentation^81–86^, we suggest that this inefficient cleavage for the Zn^2+^ complex has structural origins (*vide infra*).

Further manual inspection of the ETD spectra indicated that, similar to the CID spectra, the low m/z range of the two ETD spectra showed the same fragment ions. Thus, Zn^2+^ was, or remained bound, only to larger fragments, some of which are shown in Supplementary Fig. S14. Table 2 summarises the Zn^2+^-bound peaks identified in the ETD spectrum and it is clear that there are fewer than for the CID spectrum, and that most pertain to large fragments. The smallest ion identified to have a Zn^2+^ ion bound was, at low intensity, [z_13_+Zn]^2+^ (Fig. S14).

**Table 2:** Summary of Zn^2+^-bound peptides identified from ETD/PTR, together with experimental and theoretical masses for the respective parent fragment ions.

Assignment of fragment	Experimental [M+H]^1+^	Theoretical [M+H]^1+^	∆mass (Da)	Experimental [M-H+Zn]^1+^	Theoretical [M-H+Zn]^1+^	∆mass (Da)
z_13_*	1648.06	1647.75	+0.31	1711.06	1711.56	−0.50
c_20_	2479.50	2479.15	+0.35	2542.50	2542.65	−0.15
c_26_	3187.68	3187.46	+0.22	3250.62	3250.96	−0.34
c_27_	3324.82	3324.52	+0.30	3388.68	3388.02	+0.66
c_28_	3439.82	3439.54	+0.28	3502.76	3503.04	−0.28
z_28_*	3410.76	3410.52	+0.24	3473.76	3474.02	−0.26
z_29_*	3547.82	3548.58	−0.76	3611.62	3612.08	−0.46
z_30_*	3676.76	3676.68	+0.08	3739.62	3740.18	−0.56
z_31_*	3812.76	3812.74	+0.02	3876.82	3876.24	+0.58
c_31_	3833.76	3833.75	+0.01	3986.82	3897.25	+0.57
z_33_*	4047.94	4047.85	+0.09	4110.82	4111.35	−0.47
z_34_*	4160.94	4160.94	0.00	4223.82	4224.44	+0.62
c_34_	4195.94	4195.92	+0.02	4258.94	4259.42	−0.48

*For the fragments labelled with an asterisk, (z + 2) ions were also observed.

This would suggest that potentially any combination of six histidines (His35, His33, His30, His27, His25 or His23) could be involved in binding. There was a significant increase in the intensity of the y_7_ ion (911.61 m/z) in the presence of Zn^2+^ (Supplementary Fig. S15). This ion is the product of a secondary cleavage reaction of a *z* fragment. Its increase for the Zn_1_-HRGP330 species suggests that the presence of Zn^2+^ in the 5+ precursor ion affects the way this fragments further. The fragmentation scheme in Fig. 5 indicates that the z_8_ ion was observed only for Zn-HRGP330, and that cleavages between His25, Glu26, His27 occurred only in the absence of Zn. Such changes in fragmentation suggest that the Zn^2+^ ion is bound in the vicinity. Thus the data support the notion that this region, which harbours a HxH motif and also includes two carboxylates (with negative charges at least in solution), is one favourable location for zinc binding.

The most striking difference between the fragmentation patterns observed for the Zn_1_ and the apo peptide, irrespective of the fragmentation technique, concerned the central region around Gly16: Whilst for the apo precursor ion, all expected cleavages were observed in both CID and ETD spectra, no cleavages were observed between Pro14 and His17 when the Zn-bound species was fragmented by CID. In the ETD spectrum, the overall number of cleavages between Glu11 and His20 was reduced from six to two (Fig. 5). Clearly, the presence of Zn^2+^ in the precursor ion has affected the fragmentation efficiency in this region most dramatically. We note that this region also contains two HxH motifs.

Notably, a low degree of fragmentation is a commonly observed behaviour for folded proteins subjected to ECD or ETD under native ESI-MS conditions^36^. It is plausible that the lack of fragmentation is due to the middle part of Zn_1_-HRGP330 adopting a more rigid structure that is less susceptible to fragmentation. This stabilisation by Zn^2+^ binding also seems to provide limited protection from CID. This notion is consistent with the conclusions from ion mobility MS data which indicated that the HRGP330 structure becomes more compact when Zn^2+^ binding occurs.

Finally, given that fragments from both N-terminal and C-terminal ends of the molecule were observed to have a corresponding Zn^2+^-bound fragment in either CID (e.g. b_11_ and y_8_) or ETD (e.g. c_20_ and z_13_), it is likely that several sites are partially occupied when Zn^2+^ is present in sub-stoichiometric amounts. In summary, although some sites may be more favourable than others, it is clear that there is not a single preferred binding site for Zn^2+^ on HRGP330, but that Zn^2+^ binding in various locations modulates or decreases fragmentation.

## Discussion

This work has investigated the Zn^2+^ binding properties of a biologically active peptide from the histidine-rich region of HRG. Using native ESI-MS, up to six Zn^2+^ ions were observed to bind to HRGP330. An NMR approach was used to investigate structural changes upon Zn^2+^ binding. The uniform broadening of all His side-chain and most backbone resonances upon addition of a single molar equivalent of Zn^2+^, suggested that there is no single preferred binding site in HRGP330, and that the system contains several species in intermediate exchange. This broadening and peptide aggregation hampered detailed conclusions regarding the structure of these species. TWIM-MS showed that Zn^2+^ binding increased conformational diversity, as indicated by the observation of broader ATDs. Moreover, the average arrival times for HRGP330 with one or more Zn^2+^ ions bound were shorter than for the apo-peptide. These observations indicate that Zn^2+^ coordination does not stabilise one particular structure, even though the conformations of zinc-bound species are more compact.

Top-down MS/MS under native ESI conditions was explored as a tool to study whether preferred locations for Zn^2+^ binding could be discerned. The apo-form of HRGP330 could be fragmented by both CID and ETD with high levels of sequence coverage, most likely due to its flexible and extended conformation which allows it to fragment readily. When Zn^2+^ was bound to HRGP330, cleavage efficiency was markedly reduced in both cases, indicating that the Zn^2+^-induced conformational changes and compaction detected by TWIM-MS rendered it less amenable to fragmentation. Nevertheless, Zn^2+^-bound fragments were identified in both CID and ETD data by their mass shift from the corresponding zinc-free fragments and their isotopic distribution. The analysis of fragments and that of missing cleavages indicated that the metal ion bound to several different regions of the peptide, providing direct evidence for the suggestion that there is not a single clearly preferred binding site. This diversity in speciation may at least partially explain the broadness of the ATDs in TWIM-MS, as well as the appearance of the ^1^H NMR data: The different species have different conformations, and it is likely that Zn^2+^ ions can move between sites of similar affinity on an intermediate (NMR) timescale. Taking into account the fairly fast ligand exchange rates of Zn^2+^, this is consistent with the modest Zn^2+^ affinity reported for mammalian HRGs, with reported dissociation constants lying in the low micromolar range^30,87,88^. It is most probable that HRGP330 (and by inference HRG and many other proteins with His-rich regions) remains disordered also in the presence of Zn^2+^. Collectively, these multiple flexible metal-binding sites increase, statistically, the affinity of HRGP330 and similar protein regions for Zn^2+^ ions whilst simultaneously providing a Zn^2+^-binding module with high kinetic lability. This situation is reminiscent of metallothioneins: these predominantly intracellular Cys-rich proteins are also structurally disordered in their apo form, and combine high thermodynamic stability with high kinetic lability of metal binding – with the important difference of forming well-ordered structures in the presence of Zn^2+^ ^89^. Zn^2+^ affinity constants for metallothioneins are about six orders of magnitude larger than those of His-rich sequences. It may be suggested that the affinity of the various proteins are matched to the typical Zn^2+^ concentrations they need to deal with or operate at. Basal free Zn^2+^ concentrations in extracellular media are in the low nanomolar range^90^, and there will be little formation of HRG-Zn^2+^ complexes. However, free Zn^2+^ concentrations are subject to considerable temporal and spatial fluctuations and can rise to tens or hundreds of micromolar. These fluctuations are not random, but highly specific signals that (co-)regulate physiological processes^91^. In the case of HRG and its HRR-containing proteolytic fragment (Fig. 1), there is strong evidence that the regulatory zinc signals stem from platelets^92^. Their activation triggers the secretion of large quantities of Zn^2+^. In this way, the recruitment of platelets to sites of injury such as tumours and wounds, followed by their activation, generates a Zn^2+^-rich microenvironment^92^. This has implications for angiogenesis^15^ in malignant tumours^93^ and physiological haemostasis^29,30,94,95^. In both processes, Zn^2+^ binding to HRG, or some of its histidine-rich derivatives, increases the affinity to poly-anionic biomolecules, specifically heparin and cell surface-bound heparan sulfate. It is evident that the addition of positively charged Zn^2+^ ions to neutral His residues has the potential to greatly enhance binding to negatively charged heparin/heparan sulfate via electrostatic interactions, but previous work has shown that the role of Zn^2+^ goes beyond the provision of positive charge.

**Figure 6:**
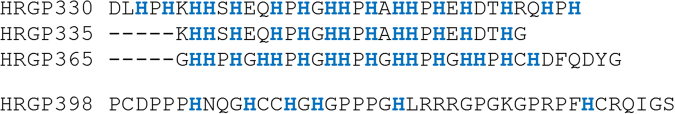
Comparison of the amino acid sequences of HRGP330 with active (HRGP335) and inactive (HRGP365 and HRGP398) peptides derived from the proteolytic fragment of HRG^33^. All His residues present in the active HRGP335 are conserved in the inactive HRGP365, which contains two additional His residues in the equivalent region. The His/Cys/Pro-rich peptide HRGP398 was also inactive.

The zinc-dependent interaction of the HRR-containing proteolytic fragment and some of its derivatives with heparan sulfate on the surface of endothelial cells alters focal adhesion and inhibits their chemotaxis, a crucial process in angiogenesis^32^. Several peptides derived from the HRR have been tested for heparin and heparan sulfate binding ability, but only HRG330 and a shorter fragment HRGP335, which lacks five residues from the N-terminus and four from the C-terminus, and has an Arg378Glu substitution (Fig. 6), were able to bind heparin with similar affinity to full-length HRG and show anti-angiogenic activity. Even shorter peptides retained activity in chemotaxis assays^34^. In contrast, the highly similar HRGP365 (Fig. 6) has much lower heparin-binding ability than HRGP330 and is unable to inhibit angiogenesis. It therefore had been postulated that Zn^2+^ would elicit a structural change or stabilise a conformation that favours recognition of heparan sulfate or heparin^15,34^. Our finding that Zn-bound structures are overall more compact than apo-HRGP330 are broadly in line with these suggestions, but the increased conformational diversity observed upon Zn binding, and simultaneous occurrence of multiple configurational species emphasise the importance of structural dynamics.

Typically, the binding and activity assays mentioned above were carried out in the presence of excess Zn^2+^ (e.g. *ca*. 5–10-fold) under physiologically relevant conditions^33,34,92^. Based on our data, it can be expected that at least 5 binding sites in HRGP330 would have been saturated. Based on literature findings and our observations, it is possible that in the Zn_5_ species, different Zn ions fulfil different purposes – provision of positive charge on the one hand, and aiding in achieving a conformation favourable for interaction with particular heparan sulfate sub-structures on the other.

Haemostasis, the first step in wound healing, is dynamically regulated by Zn^2+^ ^29^, again with platelets as central players. Several lines of evidence indicate that HRG is a central part of this dynamic regulation^30,94,95^. Zinc-mediated HRG-heparin interactions promote blood clotting, as they decrease anti-coagulatory heparin/anti-thrombin complex formation^29^. It is evident that such interactions must be transient, in order to enable eventual stopping of the clotting process. Although the effects of Zn^2+^ on haemostasis are much more complex and also include anti-coagulatory and fibrinolytic activities^29^, it can be envisaged that subsequent removal of Zn^2+^ from the vicinity of the clot, for example by binding to highly abundant serum albumin, is facilitated by the high kinetic lability of HRG-bound Zn^2+^.

Finally, it may be added that His-rich peptides and proteins and their interactions with Zn^2+^ are well suited to providing a fairly strong, yet multifunctional^3,6^ adaptor module. It is thus no surprise that HRG has been named the “Swiss army knife of mammalian plasma”^13^.

## Methods

### Materials

The HRGP330 peptide was obtained from the Keck Biotechnology Research Facility (Yale University, CT, USA). The peptide sequence is DLHPHKHHSHEQHPHGHHPHAHHPHEHDTHRQHPH with additional modifications of an acetylated N-terminus and amidated C-terminus. Analytical grade ammonium acetate, deuterated Tris, sodium chloride, zinc acetate and zinc chloride were obtained from Sigma-Aldrich (Poole, UK).

### Purification of HRGP330

Further purification of a crude HRGP330 preparation was achieved using reverse-phase high performance liquid chromatography (RP-HPLC) on an Agilent 1100 instrument. Approximately 5 mg of crude peptide was solubilised in 4:1 water:acetonitrile containing 0.1% TFA and injected onto a Jupiter Proteo 90 Å column (Phenomenex, Macclesfield, UK). The peptides were eluted at 1 mL/min with a linear gradient of acetonitrile, and absorbance was monitored at 220 nm. Pooled HPLC fractions were combined in a round-bottomed flask and concentrated on a rotary evaporator for 10 minutes. The solution was aliquoted and freeze-dried, followed by storage at −20 °C until required. Due to the lack of aromatic amino acids in the peptide sequence, Scopes’ equation was used to estimate the concentration by measuring the absorbance at 205 nm^96^.

### Native ESI-MS

HRGP330 was reconstituted in 10 mM ammonium acetate (pH 7.4) and desalted using 5 mL polyacrylamide columns (Thermo Fisher Scientific, Hemel Hempstead, UK). Zinc acetate stock solutions were prepared in ultrapure water and added to HRGP330 in microlitre aliquots to achieve metal: peptide ratios ranging from 1:1 to 5:1. Positive electrospray mass spectra were acquired on a maXis^TM^ UHR-Q-TOF instrument (Bruker Daltonics, Coventry, UK) calibrated with sodium formate. A syringe pump injected the sample into the mass spectrometer at a rate of 90 µL/hr. Raw data were collected for 2 minutes over a m/z range of 500–1500. The following conditions were used for the Q-TOF mass spectrometer: Dry gas = 4.0 L/min; dry gas temperature = 453 K; funnel RF = 400 Vpp; multiple RF = 400 Vpp; ISCID = 0 eV; collision cell energy = 10 eV; collision RF = 1300 Vpp; ion cooler = 650 Vpp; transfer time = 148.4 µs. The data were averaged and deconvoluted using Bruker Compass analysis software.

### ^1^H-NMR spectroscopy

Samples of HRGP330 (0.5 mM) were prepared in 50 mM [D_11_]Tris-Cl, 50 mM NaCl, 10% D_2_0, at pH* 6.20. Additions of Zn^2+^ ions were achieved using microlitre aliquots of 50 mM ZnCl_2_. ^1^H-NMR spectra were recorded on an Avance 700 Ultrashield^TM^ spectrometer (Bruker BioSpin) which had an operating frequency of 700.13 MHz for ^1^H. Suppression of the water signal was achieved using excitation sculpting with gradients^97^. The 1D spectra were obtained at 278 K using 4k complex data points and 128 scans. In the 2D [^1^H, ^1^H] total correlation (TOCSY) and nuclear Overhauser (NOESY) experiments, spectra were acquired with 32 scans over 4k data points in the F2 dimension and 512 increments in F1. The spectral width was 13 ppm in both dimensions. A spin lock of 60 ms for the TOCSY and mixing time of 500 ms for the NOESY experiment were used. The raw data were apodised using shifted sine-bell functions and Fourier-transformed with 2k × 2k data points in F2 and F1. Baseline correction was employed in both dimensions. Spectra were processed using TOPSPIN 2.1 (Bruker BioSpin).

### Comparison of apo*-* and Zn-bound HRGP330 using TWIM-MS

Samples of HRGP330 were prepared using the same procedure as described above under “native ESI-MS”. HRGP330 (10 µM) in 10 mM ammonium acetate (pH 7.4) was injected via a nanoflow source into a Synapt HDMS G2 system (Waters Corporation, Milford, MA, USA)^98^. The instrument was operated in the positive mode with a source temperature of 363 K. The following instrumental conditions were used: backing pressure 2 mBar, capillary voltage 1.2 kV, cone voltage 40 V, helium cell gas flow 180 mL/min, IMS cell gas flow 90 mL/min, travelling-wave height 40 V and travelling wave velocity 700 m/s. A mass acquisition range of 200–1500 m/z was used. The ion mobility data were calibrated with polyalanine which had been analysed under the same conditions. Data were analysed and processed using MassLynx v4.1 (Waters).

### MS/MS experiments

MS/MS experiments were carried out using a dual ion funnel AmaZon speed ETD instrument (Bruker Daltonics) operating in positive mode. HRGP330 was infused at a rate of 6 μL/min and acquisitions were performed using a scan speed of 8100 m/z per second. The following parameters were used: trap drive = 71.8; capillary exit = 140 V; source temperature = 453 K. An isolation width of 4 m/z was used to select the ion of interest and CID was performed by applying an amplitude of 1.0 V. Fluoranthene was used as the electron transfer dissociation reagent with an ETD reaction time of 100–120 ms, followed by a proton transfer reaction (PTR) time of 75 ms. Monoisotopic peaks were assigned using the SNAP™ peak detection algorithm. Spectra were analysed using BioTools v3.2 and Sequence Editor (Bruker Daltonics).

## Electronic supplementary material

Supplementary Information
