# Supplementary material for: Native electrospray mass spectrometry approaches to probe the interaction between zinc and an anti-angiogenic peptide from histidine-rich glycoprotein
Source: Sci Rep. 2018 Jun 5;8:8646. doi: 10.1038/s41598-018-26924-1 (PMC5988744; doi:10.1038/s41598-018-26924-1)
Supplement: Supplementary file 1 — Supplementary Information [file 41598_2018_26924_MOESM1_ESM.pdf]

Supplementary information

# Native electrospray mass spectrometry approaches to probe the interaction between zinc and an anti-angiogenic peptide from histidine-rich glycoprotein

Esther M. Martin,[a,b] Frances D. L. Kondrat,[c,d] Alan J. Stewart,[e] James H. Scrivens,[c,f] Peter J. Sadler,[a] and Claudia A. Blindauer[a]

[a]*Department of Chemistry, University of Warwick, Coventry, UK*

[b]*Medimmune, Cambridge, UK*

[c]*School of Life Sciences, University of Warwick, Coventry, UK*

[d] *Immunocore Ltd, Abingdon, UK*

[e]*School of Medicine, University of St Andrews, St Andrews, UK*

[f] *School of Science, Engineering and Design, Teeside University, Middlesbrough, UK*

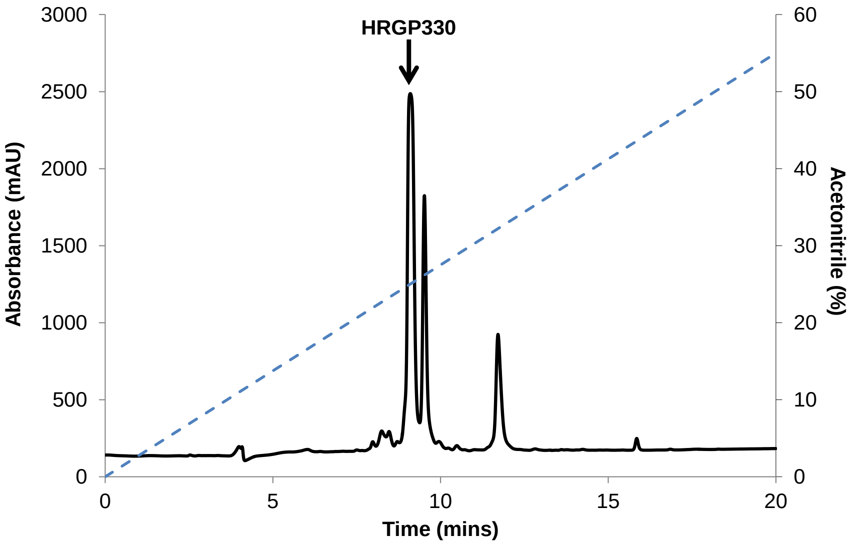


**Fig. S1 Purification of HRGP330 by RP-HPLC** Example of a chromatogram obtained from loading crude HRGP330 onto a Jupiter Proteo 90A column using an Agilent 1100 instrument. The blue line shows an increasing gradient of acetonitrile containing 0.1 % TFA. HRGP330 eluted at 9.2 min shown by the most abundant peak in the chromatogram

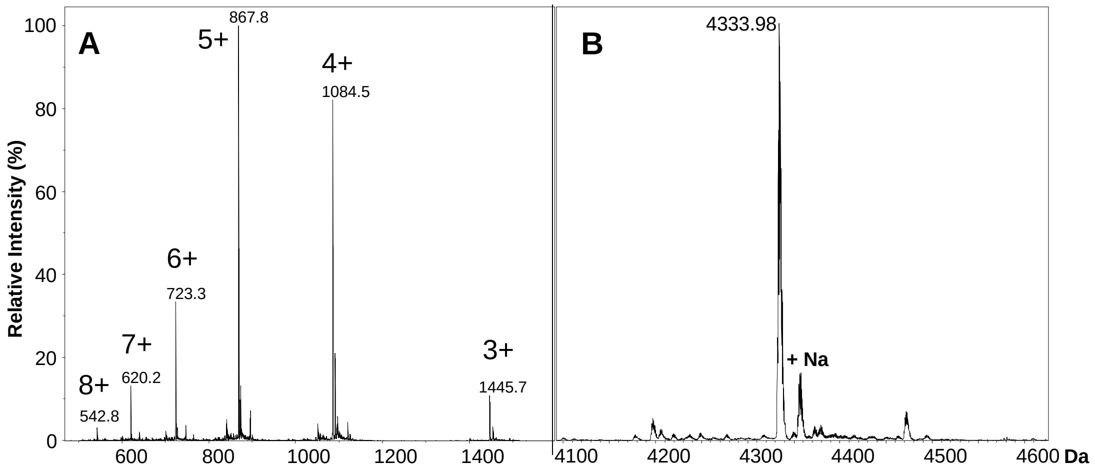


| Charge state | Experimental *m/z* | Theoretical *m/z* | ∆mass (Da) |
|---|---|---|---|
| 3+ | 1445.00 | 1445.00 | **0.00** |
| 4+ | 1083.99 | 1084.00 | **0.01** |
| 5+ | 867.39 | 867.40 | **0.01** |
| 6+ | 722.99 | 723.00 | **0.01** |
| 7+ | 619.85 | 619.86 | **0.01** |
| 8+ | 542.49 | 542.50 | **0.01** |

**Fig. S2 Identification of purified HRGP330 ESI-MS** HPLC fractions were injected directly into the microTOF mass spectrometer to produce the spectrum A) Charge state envelope B) Deconvoluted mass spectrum. Table shows the exact m/z values for the mono-isotopic peaks, obtained from a maXis high resolution mass spectrometer, confirming the peptide identity.

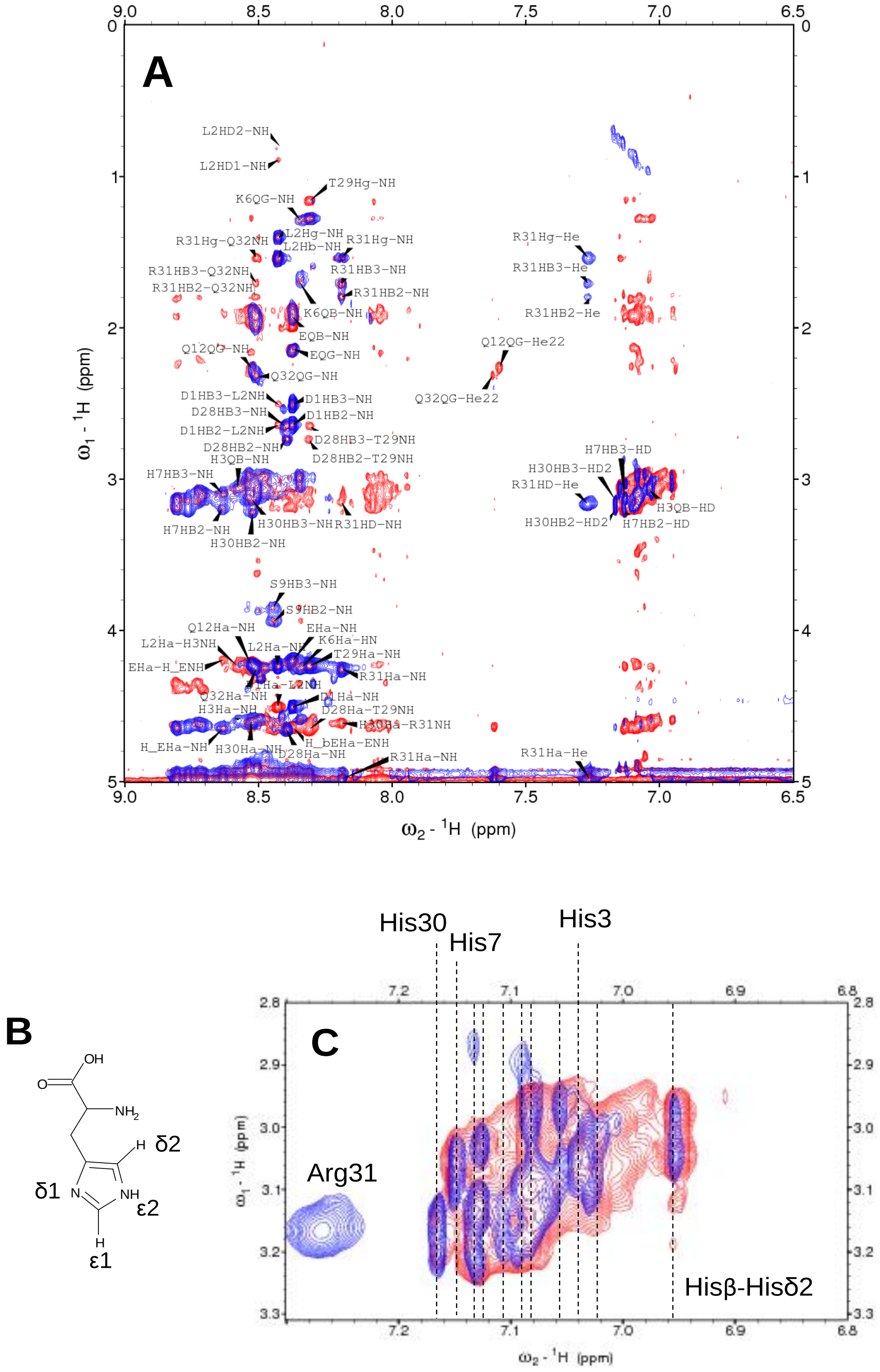


**Figure S3 Fingerprint region of 700 MHz 2D [$^1$H,$^1$H] NMR TOCSY (blue) and NOESY (red) spectra of HRGP330.** A) Full fingerprint region showing assigned peaks. B) Chemical structure of His showing nomenclature of resonances. C) 11 His residues can be distinguished from their Hisβ-Hisδ2 crosspeaks, each indicated with a dashed line. Sample concentration was ~0.5 mM in 50 mM Tris[$D_{11}$], 50 mM NaCl with 10% $D_2O$. The pH* was 6.20 and the spectra were obtained at 278 K.

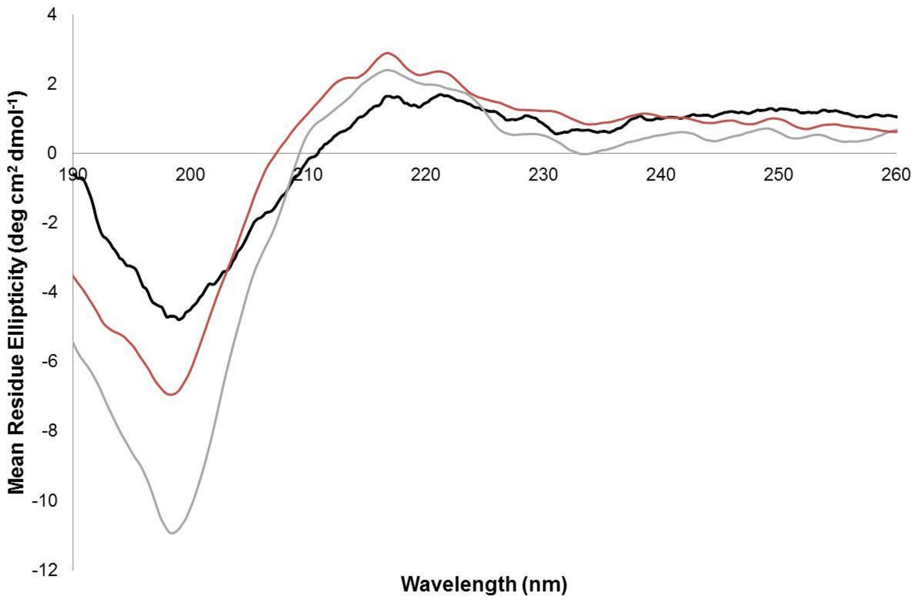


**Figure S4 Far-UV region of the CD spectrum of HRGP330 in the presence and absence of $Zn^{2+}$.** *Black* line: HRGP330 (11 $\mu$M in 10 mM potassium phosphate, pH 7.4). *Red* line: after addition of 2 mol. equiv. $ZnCl_2$. *Grey* line after addition of 5. mol. equiv. $ZnCl_2$.

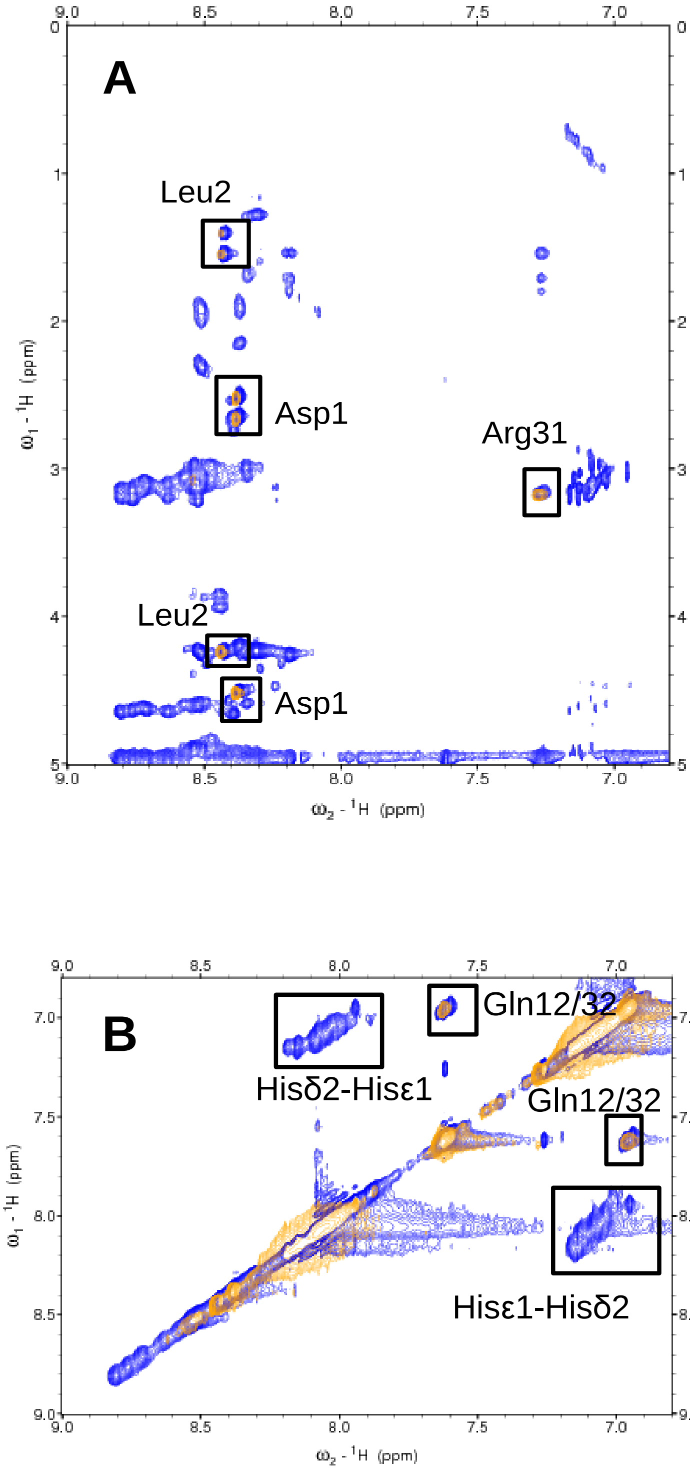


**Figure S5 Effect of $Zn^{2+}$ on 700 MHz 2D TOCSY $^1H$ NMR spectrum of HRGP330.** A) Fingerprint region B) Aromatic region. The TOCSY spectrum of Zn-HRGP330 (*orange*) is overlaid onto the TOCSY spectrum of apo-HRGP330 (*blue*). ~0.5 mM samples were prepared in 50 mM Tris[$D_{11}$], 50 mM NaCl with 10 % $D_2O$ at pH* 6.20 and 278 K. Whilst the $NH_2$ amide proton crosspeaks from non-coordinating glutamines are largely unaffected, all histidine Hδ2/Hε1 crosspeaks are broadened beyond detection even at a ratio of 1 Zn:17 His. This is likely a consequence of direct interactions of the zinc ions with all 17 His residues within the NMR (exchange) timescale.

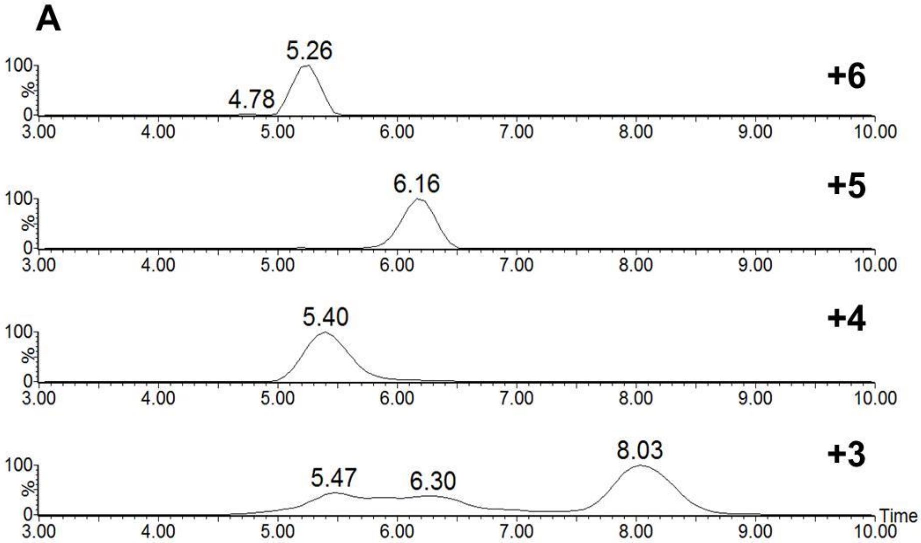


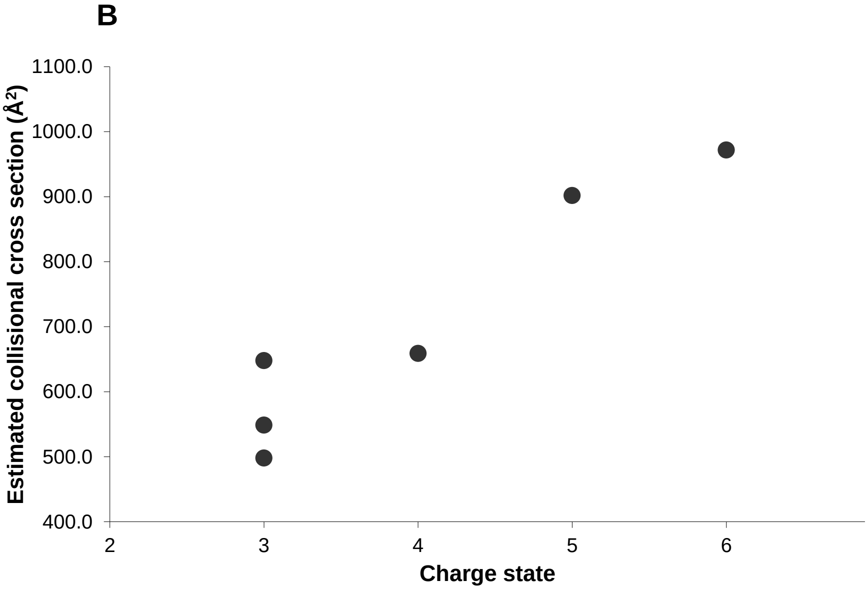


**Figure S6 ATDs and estimated collisional cross sections of the charge states observed for apo-HRGP330.** A) ATDs for the charge states observed. B) Estimated collisional cross sections calculated from the ATDs. The samples were 10 $\mu$M HRGP330 in 10 mM ammonium acetate (pH 7.4). There are 3 points for the 3+ charge state representing the 3 different conformations observed. The estimated cross sections were the same across two datasets.

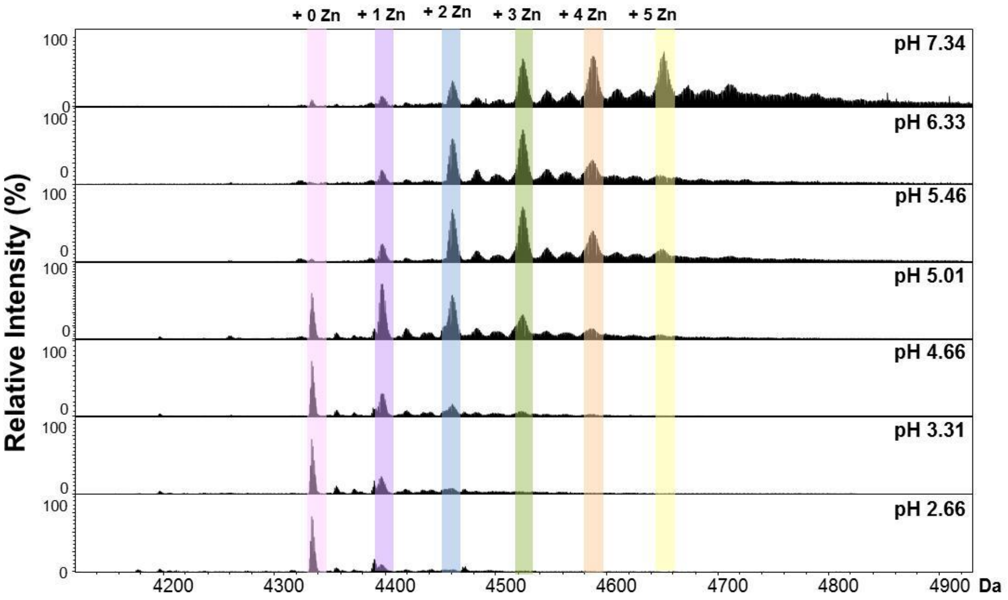


**Figure S7 pH titration of $Zn_5$-HRGP330.** $Zn_5$-HRGP330 (20 $\mu$M) was titrated with microlitre additions of 1% acetic acid followed by analysis using ESI-MS. The amount of $Zn^{2+}$ bound drops off significantly below pH 5, although minor amounts of the $Zn_1$ species are discernible even at pH 2.66.

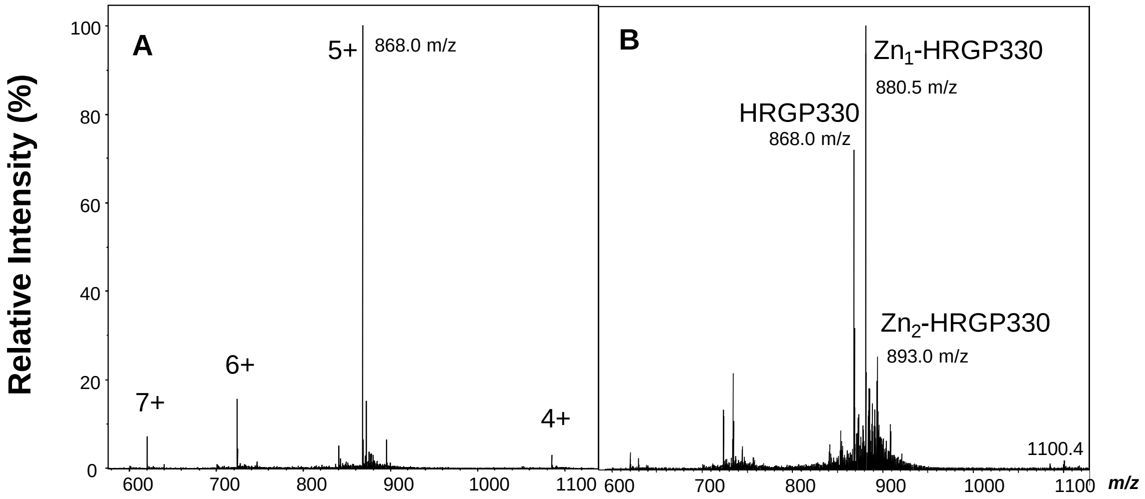


**Fig. S8 ESI-MS spectra of intact HRGP330 prior to MS/MS experiments** A) Charge-state envelope for apo-HRGP330 in 10 mM ammonium acetate (pH 7.40). B) Addition of 1 mol equiv zinc acetate yielded metallated HRGP330. The two peaks at 868 and 880.5 m/z were isolated for the top-down experiment.

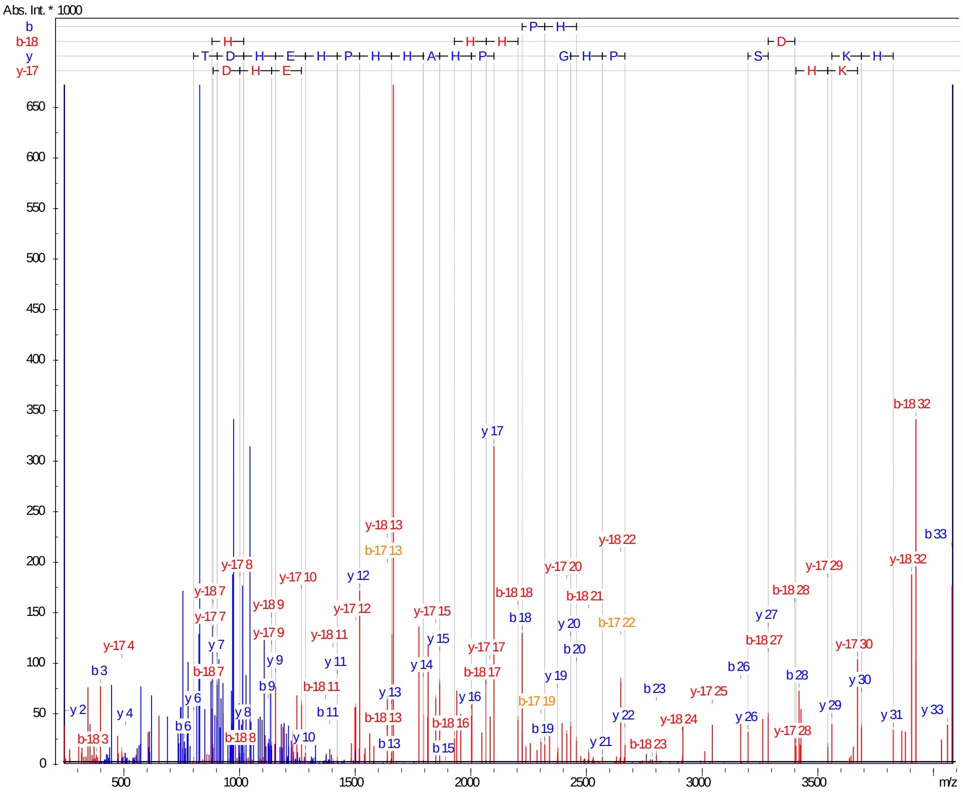


**Figure S9 CID spectrum of apo-HRGP330 (868 m/z).** Biotools v3.2 was used to assign b and y fragment ions. Simple b- and y-ions are shown and labelled in blue. Ions labelled “-17” (orange for b-ions, red for y-ions) and “-18” (red) indicate ions resulting from neutral losses of $NH_3$ or $H_2O$, respectively. The resulting fragmentation scheme is shown in Figure 5 in the manuscript.

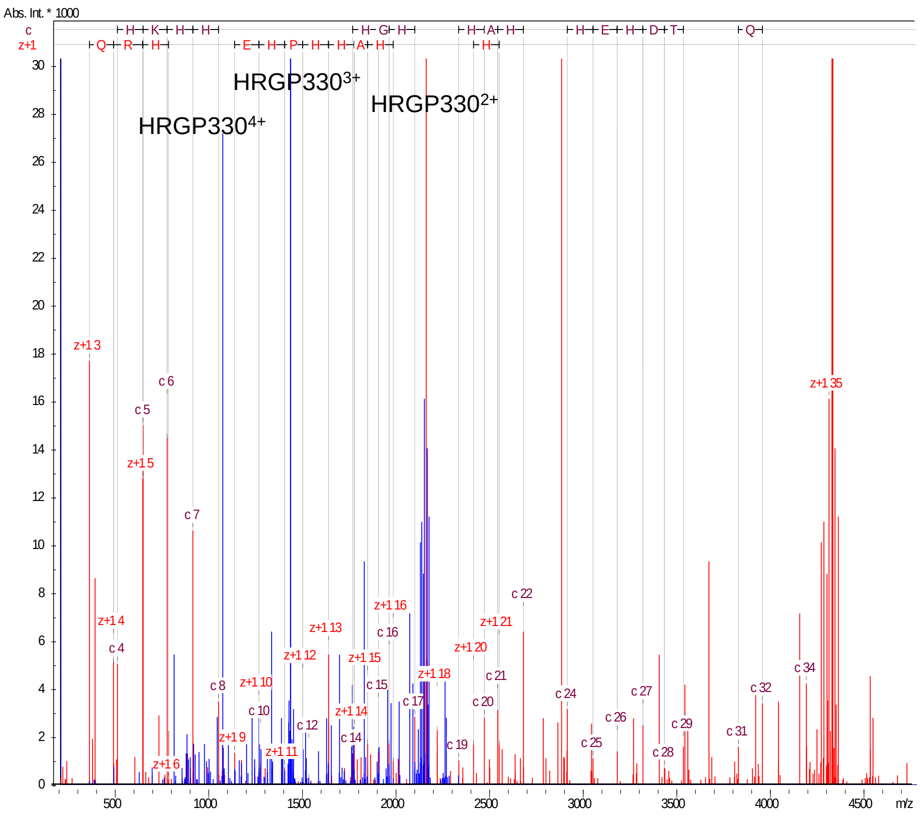


**Figure S10 ETD spectrum of apo-HRGP330 (868 m/z).** Biotools v3.2 was used to assign c (labelled in purple) and z+1(labelled in red) fragment ions. The resulting fragmentation scheme is shown in Figure 5.

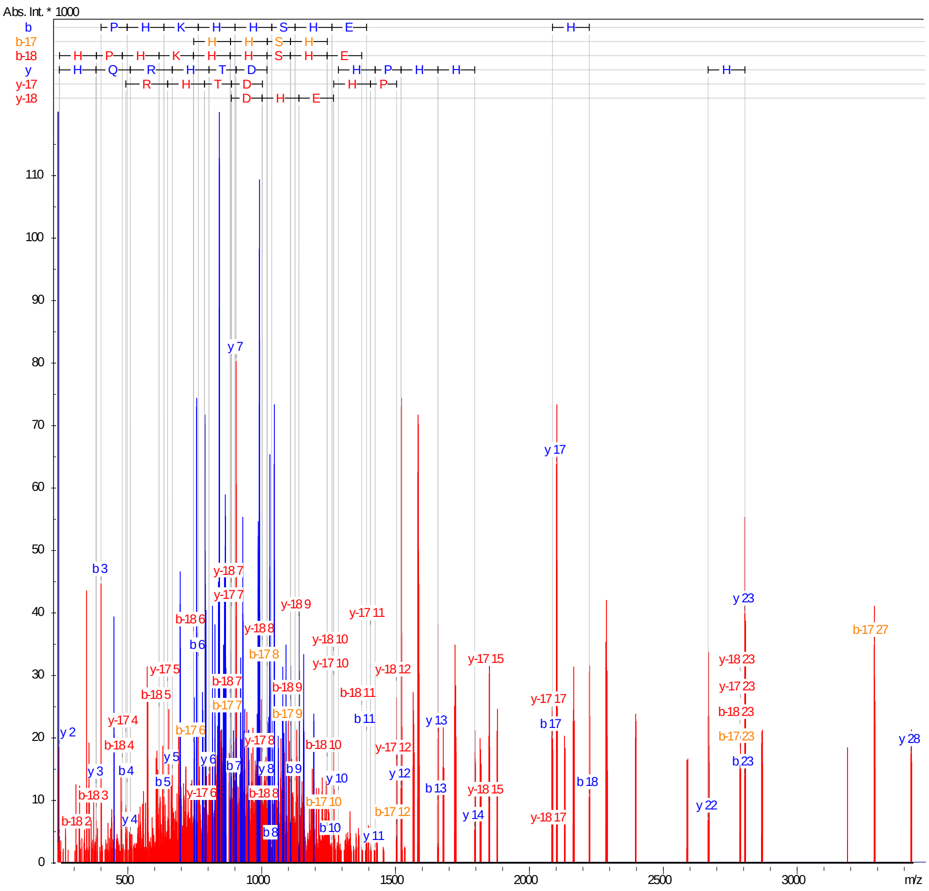

**Figure S11 CID Spectrum of $Zn_1$-HRGP330 (880.5 m/z).** Biotools v3.2 was used to assign b and y fragment ions. Simple b- and y-ions are shown and labelled in blue. Ions labelled “-17” (orange for b-ions, red for y-ions) and “-18” (red) indicate ions resulting from neutral losses of $NH_3$ or $H_2O$, respectively. The resulting fragmentation scheme is shown in Figure 5 in the manuscript.

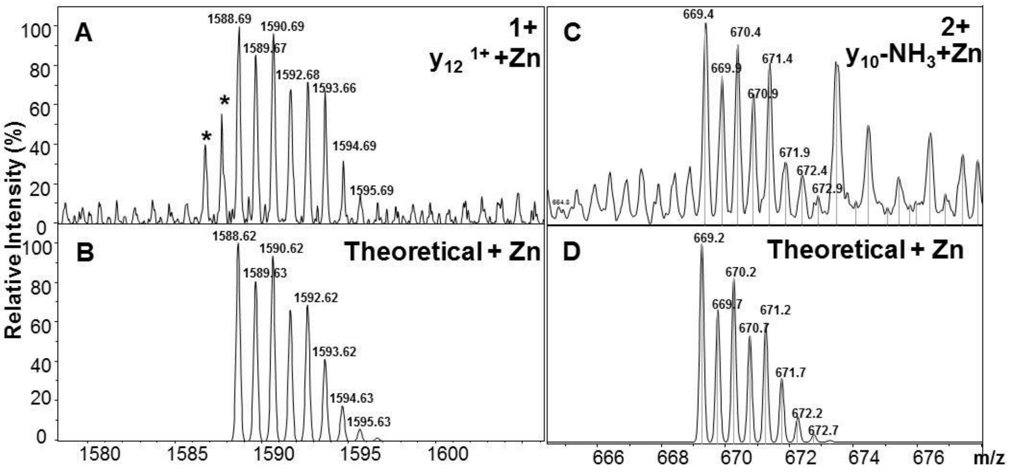


**Figure S12 Comparison of experimental and theoretical isotopic patterns for the $Zn^{2+}$ adducts of $y_{12}$ and $y_{10}$ generated from CID.** A) Experimental isotopic distribution of the $y_{12}^{1+}$ ion with $Zn^{2+}$ bound B) Theoretical isotopic distribution of the $y_{12}^{1+}$ ion with $Zn^{2+}$ bound. C) Experimental isotopic distribution of the $y_{10}^{2+}$ ion with a loss of $NH_3$ and $Zn^{2+}$ bound D) Theoretical isotopic distribution of the $y_{10}^{2+}$ ion with a loss of $NH_3$ and $Zn^{2+}$ bound. The peaks marked with * are contaminating peaks.

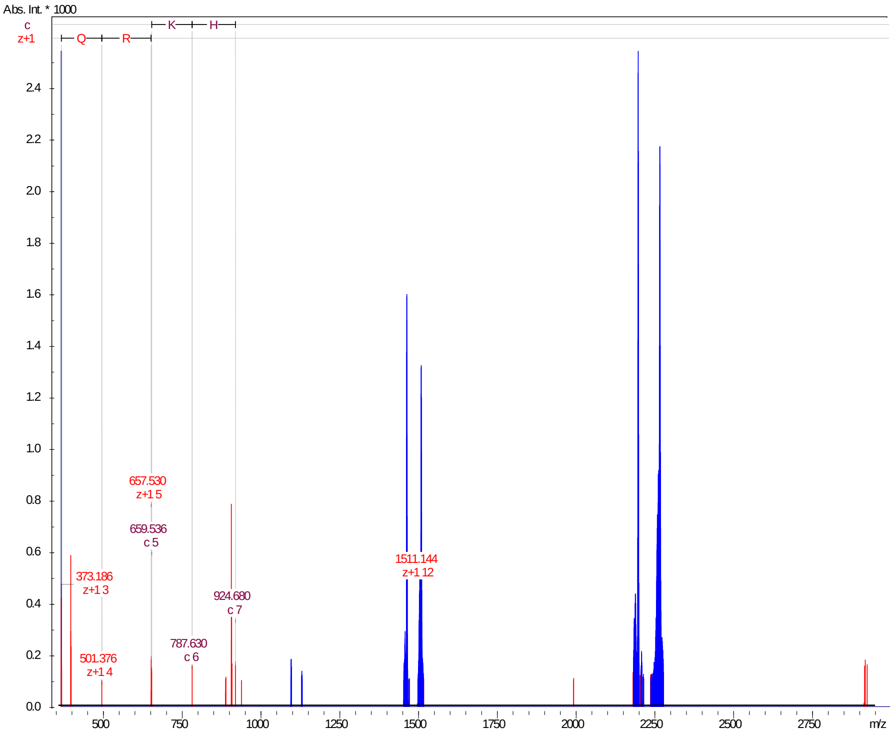


**Figure S13 ETD Spectrum of $Zn_1$-HRGP330.** Most of the fragment peaks were below the intensity threshold to be recognised by the Biotools software. Therefore, the raw data were manually analysed to obtain the fragmentation scheme shown in Figure 5.

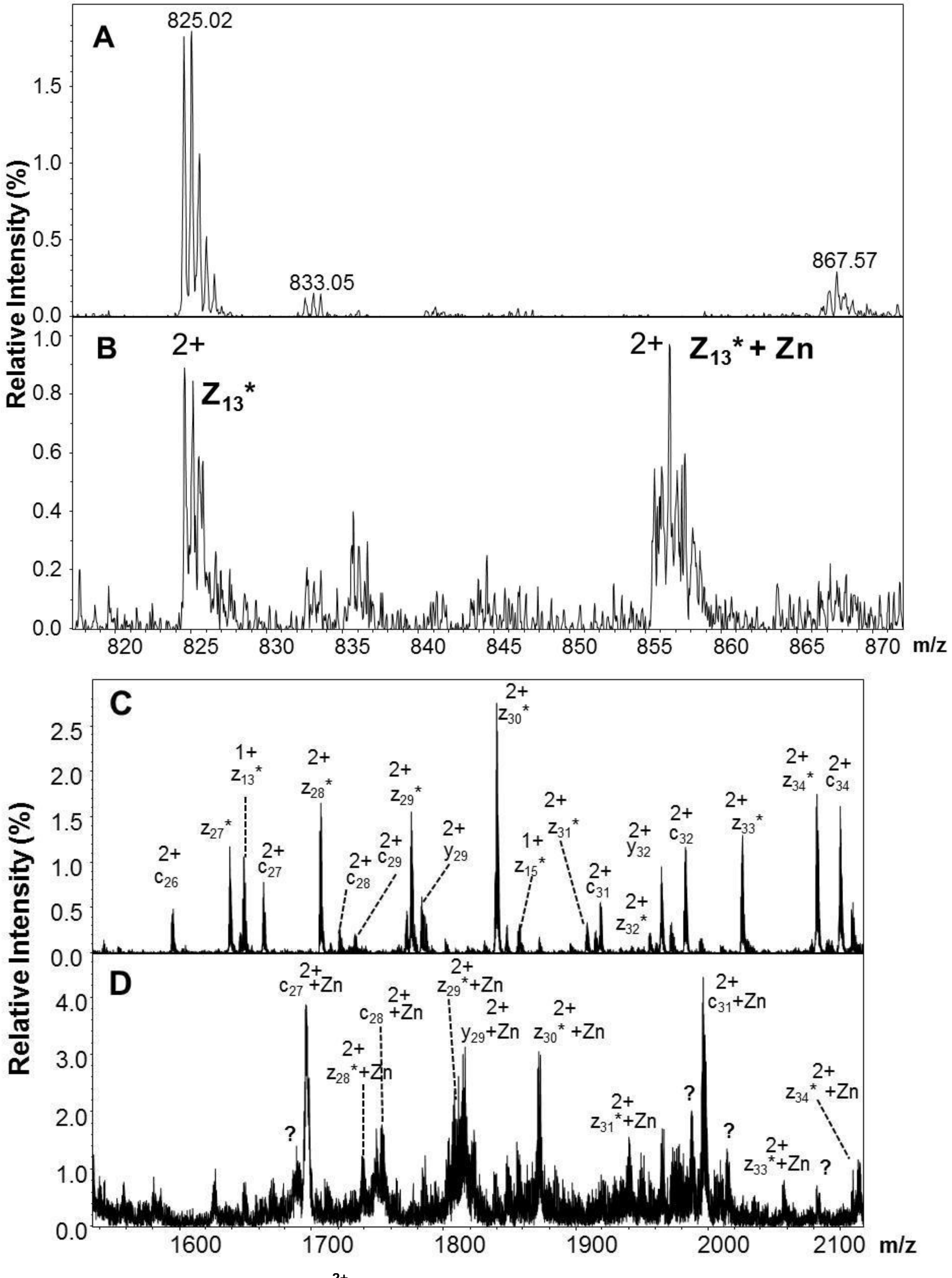


**Figure S14 Identification of $Zn^{2+}$-adducts in the ETD spectrum of $Zn_1$-HRGP330** A) ETD spectrum of apo-HRGP330 (868 m/z) showing the $[z_{13}+2]^{2+}$ ion B) Observation of a $Zn^{2+}$ ion bound to $z_{13}$ (fragmentation of 880.5 m/z) C) The ETD spectrum of 868 m/z shows a number of clearly resolved peaks between 1500-2100 m/z D) The ETD spectrum of 880.5 m/z shows a number of peaks identified as $Zn^{2+}$-adducts, although the spectrum is much noisier. Further unassigned weak peaks that appear to contain $Zn^{2+}$ are marked with a **?.**

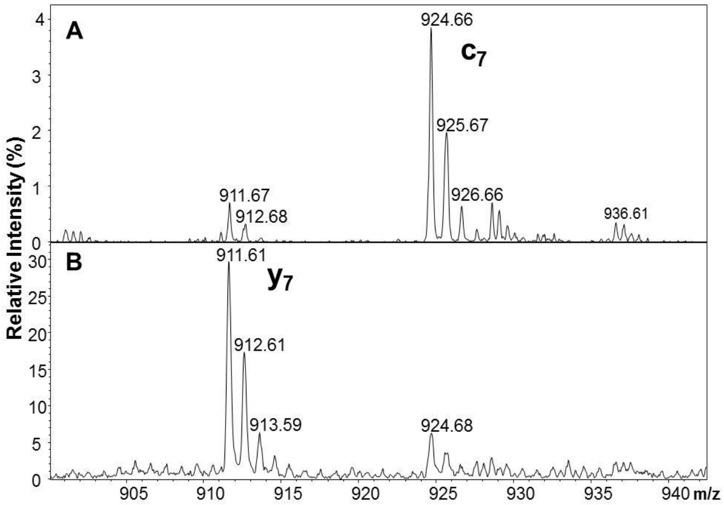


**Figure S15 $Zn^{2+}$ binding to HRGP330 has an effect on the relative intensity of different ions produced by ETD.** A) $c_7$ ion observed in the ETD spectrum of 868.0 m/z B) $y_7$ ion observed in the ETD spectrum of 880.5 m/z. The y ion is produced through a secondary reaction.
